# Inhibitory Activity and Docking Analysis of Antimalarial Agents from* Stemona* sp. toward Ferredoxin-NADP+ Reductase from Malaria Parasites

**DOI:** 10.1155/2018/3469132

**Published:** 2018-08-26

**Authors:** Pratiwi Pudjiastuti, Ni Nyoman T. Puspaningsih, Imam Siswanto, Much. Z. Fanani, Yoko K. Ariga, Toshiharu Hase, Satyajit D. Sarker, Lutfun Nahar

**Affiliations:** ^1^Department of Chemistry, Faculty of Science and Technology, Airlangga University, Surabaya 60115, Indonesia; ^2^Laboratory of Proteomics, Institute of Tropical Disease, Airlangga University, Surabaya 60115, Indonesia; ^3^Graduate School of Sciences and Technology for Innovation, Department of Agriculture, Yamaguchi University, Yamaguchi 753-8515, Japan; ^4^Institute of Protein Research, Osaka University, 3-2 Yamadoaka, Suita-shi, Osaka 656-0871, Japan; ^5^Medicinal Chemistry and Natural Products Research Group, School of Pharmacy and Biomolecular Sciences, Liverpool John Moores University, James Parsons Building, Byrom Street, Liverpool L3 3AF, UK

## Abstract

Ferredoxin-NADP^+^ reductases (FNRs, EC 1.18.1.2) were found in the plastids of* Plasmodium *and have been considered as a target for the development of new antimalarial agents. Croomine,* epi*-croomine, tuberostemonine, javastemonine A, and isoprotostemonine are isolated alkaloids from the roots of* Stemona *sp. and their inhibitory effect on FNRs from* Plasmodium falciparum (Pf*FNR) was investigated. Croomine showed the highest level of inhibition (33.9%) of electron transfer from* Pf*FNR to* Pf*Fd, while tuberstemonine displayed the highest level of inhibition (55.4%) of diaphorase activity of* Pf*FNR. Docking analysis represented that croomine is located at the middle position of* Pf*FNR and* Pf*Fd. Croomine from* S. tuberosa* appeared to have potential as an antimalarial agent.

## 1. Introduction

Malaria is still considered as a threat in the world, especially in Asia and Africa. Almost 97 countries in the world are malaria endemic. More than 3.3 billion people (almost 50% of the world's population) are at risk of malaria with two* Plasmodium *species responsible for the majority of infections:* P. falciparum *and* P. vivax *[1]. Many antimalarial agents, such as chloroquine, pyrimethamine, quinine, proguanil, sulfadoxine, and artemisinin have been used for malarial therapy. Artemisinin, the first-line antimalarial drug has been used against* P. falciparum* in Cambodia, Thailand, Vietnam, and Myanmar, where artemisinin-resistant parasites have already been identified [1]. In Papua, Indonesia, resistance to artemisinin derivatives, artesunate and dihydroartemisinin, and semisynthetic 10-alkylamino-artemisinin derivatives, artemisone and artemiside, has also been observed in* P*.* falciparum *[2]. Ratcliff, et al. (2007) studied 143 patients (103 and 40 patients) who were treated with chloroquine-sulfadoxine and chloroquine, respectively. The failure rate at 28 and 42 days was 65% and 48% for* P*.* falciparum *and* P. vivax, *respectively [3]. Pyrimethamine and sulfadoxine can inhibit dihydrofolate reductase (DHFR) and dihydropteroate synthase (DHPS) enzymes. These enzymes undergo gene mutations causing the resistance of antimalarial antifolate [4]. Double and quadruple mutations in the* P. vivax *dihydrofolate reductase (*Pv*DHFR) found in Papua* P. vivax* isolates were 46 and 18%, respectively [5].

*Plasmodium* sp. has a unique organelle, called apicoplast, and it possesses metabolic pathways, which is not found in human host [6, 7]. The apicoplast was firstly found in* P. falciparum* and* Toxoplasma *[8]. The function of plastid (apicoplast) in Apicomplexa is unclear, but many studies suggested its roles in the biosynthesis of fatty acids, haem, isoprenoids, and Fe-S cluster, based on the analysis of genome sequence. More importantly, the metabolism of apicoplast is necessary for the survival of the* Plasmodium *[6, 9, 10].

Ferredoxin (Fd) and Fd-NADP^+^ reductase (FNR) in apicoplast constitute a redox system [11, 12] that is homologous to those of plant chloroplast and algae, especially green and red algae [8, 13].The involvement of apicoplast Fd has been indicated in the reactions of an Fe-S cluster assembly [14] and isoprenoid biosynthesis pathways [10, 15], fatty acid desaturation [5], and heme oxygenation [16].

In a previous study,* in silico* analysis showed that croomine and* epi*-croomine could form hydrogen bonds with the amino acid residue of Ala9 of the DHFR enzyme [17]. DHFR can be found in human and* Plasmodium*, but FNR is found only in* Plasmodium*. Fd and FNR were cloned from* P. falciparum *and their interactions were characterized [9, 18]. There has been no report on plant-alkaloid inhibiting FNR available to date. Here, we describe the inhibitory effect of alkaloids from the roots of* Stemona *sp. on the* Pf*Fd/FNR redox system, and the results of their docking analysis.

## 2. Materials and Methods

### 2.1. Isolation of Alkaloid from Stemona Species

The dry roots of* S. tuberosa* were cut in small size and ground into powder and extracted using ethanol. The filtrate was evaporated under vacuum and extracted by acid based method using 5% of HCl and conc of NH_4_OH. Water fraction was partitioned using dichloromethane (DCM) and evaporated. The crude alkaloid was separated using column chromatography under silica gel as stationary phase and DCM-methanol as mobile phase with polarity gradient elution. The alkaloids were purified using preparative chromatography to give tuberostemonine, croomine, and* epi*-croomine (*epi*-10-hydroxycroomine or 3-*epi*-tuberospironine A) [19].

**Figure 1:**
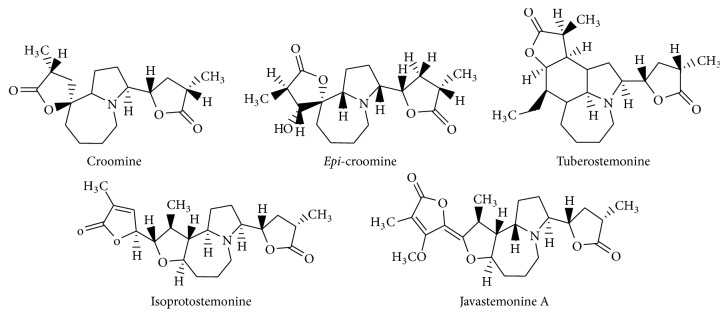
Structure of alkaloids from the roots of* Stemona* sp.

Isolation of isoprotostemonine and javastemonine A from* S. javanica *utilized the same method as described for the isolation of alkaloids from* S. tuberosa*. Previous study exhibited that tuberostemonine, croomine, and* epi*-croomine were treated to antifolate target of DHFR enzymes. Isoprotostemonine and javastemonine A from* S. javanica *were carried out against* P. falciparum* multidrug resistant and wild type parasites at Medical Molecular Research Unit, Thailand Science Park, Pathumthani, Thailand [20]. The structures of alkaloids were shown in Figure 1. Five alkaloids were made as 1000 ppm stock solution (in 0.2% of DMSO in suspension of phosphate buffer at pH 7.0). The final alkaloids solution concentration was 100 ppm.

### 2.2. Preparation of Recombinant PfFNR and PfFd

Production and purification of recombinant* Pf*Fd and* Pf*FNR were performed as reported by Kimata-Ariga et al. [9].

### 2.3. Enzymatic Activity Analysis

Enzyme activity of* Pf*FNR was measured using a grating microplate reader (model SH-1000Lab, CORONA, Japan). Diaphorase activity of FNR with DCPIP as an electron acceptor (Scheme 1) was measured as reported by Onda [21] with modification in the reaction mixture of 50 mM Tris-HCl (pH 7.5), 1 mM MgCl_2_, 0.2 mM DCPIP, and 5 nM FNR. The reaction was initiated by adding 5-100 *μ*M of NADPH and the decrease of the absorbance was monitored for 5 min with 10-second intervals at 600 nm. The activity of NADPH-dependent electron transfer from FNR to Fd (Scheme 2) was measured using cytochrome* c* (cyt* c*) as an arbitrary electron acceptor as reported by Onda [21] with modification in the reaction mixture of 50 mM Tris-HCl (pH 7.5), 100 mM NaCl, 200 *μ*M cyt*c*, and 10 nM* Pf*FNR. Electron transfer reaction was initiated by adding 0.5-40 *μ*M* Pf*Fd and 100 *μ*M NADPH, and the absorbance was monitored for 5 min with 10-second intervals at 550 nm. The kinetic parameters for the Michaelis constant (*K*_*m*_) for these reactions were determined.

**Scheme 1 (NADPH *➝*FNR*➝* DCPIP).**

Scheme 1 is used for measuring the NADPH-dependent catalytic activity of FNR.

**Scheme 2 (NADPH *➝*FNR*➝*Fd *➝* cyt*c*).**

Scheme 2 is used for measuring the NADPH-dependent electron transfer activity from FNR to Fd.

### 2.4. Inhibition Assay

Inhibition of diaphorase activity of* Pf*FNR was measured in the reaction of Scheme 1 as described above, by the additions of each alkaloid at 100 ppm, NADPH at 100 uM, and 5 nM FNR. Inhibition of the electron transfer from* Pf*FNR to* Pf*Fd was measured in the reaction of Scheme 2 as described above, by the additions of each alkaloid at 100 ppm and* Pf*Fd at 1 *μ*M. A 5 mM solution of DMSO was used as a control.

### 2.5. Docking Procedures

Molecular docking was performed using Dock 6.8 software (http://dock.compbio.ucsf.edu/DOCK_6/index.htm) [22]. Docking studies were carried out on an Intel Core i5 2.4 GHz PC, with 16 GB memory and with Windows 10 operating system.

## 3. Ligand and Receptor Preparations

The receptor model was obtained from Protein Data Bank (PDB) server (http://www.rcsb.org/) with ID 1GAQ [23]. This complex consists of three chains, there are enzyme* Plasmodium falciparum* (*Pf*FNR), flavin adenine dinucleotide (FAD), and some crystals of water. Crystals of water were not involved in the reaction mechanism directly and were stripped out from the complex. In contrary, FAD was used as a part of the receptor. Hydrogen and charge atoms were added to* Pf*FNR and FAD complex using molecular mechanics method with AMBERff14SB force field [24]. Hydrogen atom was stripped out from* Pf*FNR and hydrogen-free* Pf*FNR was saved in pdb format to generate molecular surface of the receptor.

Ligand models were drawn using HyperChem6 software of HyperCube, Inc (http://www.hyper.com/). All the ligand models were preoptimized using MM+ molecular mechanics methods before being optimized utilizing semiempirical method AM1 to generate 3D conformation also in HyperChem 6 as final process. These optimized ligands were saved in MDL mol file format. Hydrogen and charge atoms were added and loaded using the AM1-BCC charge calculation [25] and UCSF Chimera (https://www.cgl.ucsf.edu/chimera/) [26] methods, respectively. All these protonated and charged ligands were saved in Sybil mol2 files format.

## 4. Ligands Docking

**Figure 2:**
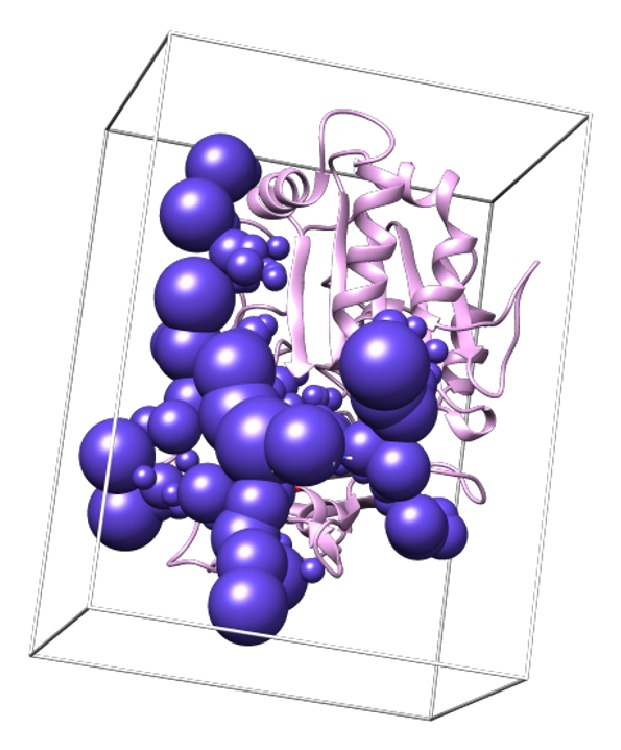
Selected cluster sphere of the receptor as the representation of the binding site is inside the grid box.

The molecular surface of the receptor was generated with the dms tools which was included in the DOCK 6.8 package program. The molecular surface was achieved by dms tool and spheres of the receptor were generated using program sphgen [27]. Binding site was represented by the largest cluster of the spheres generated of sphgen. Grid box for docking simulation was created around the binding site with extra margin 5 Å. Grid space was set in 0.2Å, while the dimension is 47.814Å x 63.571Å x 39.732Å. The location of the largest cluster of the spheres as the representation of the binding site and grid box on the receptor was shown in Figure 2. Each ligand was docked within the binding site of the receptor using Dock 6.8 resulting in binding (Grid_score) and van der Waals (Grid_vdw) energies, electrostatic interaction (Grid_es), and internal energy of the complex (Int_energy). Int_energy was determined based on force of field energy calculated.

## 5. Results and Discussion

The structures of alkaloids from the roots of* Stemona* species are shown in Figure 1.

### 5.1. Inhibition of NADPH-Dependent Catalytic Activity of PfFNR by Alkaloids from the Stemona sp.

The effect of these alkaloids from the roots of* Stemona* spp. (Figure 1) on the NADPH-dependent catalytic activity (diaphorase activity) of* Pf*FNR (Table 1) was inquired. In this reaction, the effects on the FNR catalytic activity and/or interaction with NADPH were to be considered. In the absence of the inhibitors, the* K*_*m*_ value for NADPH was 3.57 *μ*M. The inhibition assay was performed under the same conditions used in the* K*_*m*_ determination employing the following conditions: the concentration of NADPH was 100 *μ*M, the concentration of* Pf*FNR was 5 nM, and the concentration of tested compounds was 100 ppm. The % inhibition was calculated according to the following equation, and the results are listed in Table 1.

(1) %  Inhibition=dA/dtcontrol−dA/dtsampledA/dtcontrol×100%

The results showed that three out of the five tested compounds were able to inhibit the reaction by between 0.7 and 33.9%, while the other two compounds exhibited a negative inhibition, which means that these compounds accelerated the activity. Croomine alkaloid showed 33.9%, the largest inhibition, and* epi*-croomine showed lower but considerable inhibition. Isoprotostemonine and javastemonine A exhibited a negative inhibition. This might be because both alkaloids have double bonds conjugated to carbonyl group and, thus, might considerably trigger electron delocalization and stabilisation of resonance. Stabilisation of resonance in isoprotostemonine and javastemonine A, which means that these compounds accelerated the electron transfer from PfFNR to PfFd, will make* P. falciparum* parasites live because electron transfer occurs in respiratory process [28].

### 5.2. Inhibition of the Electron Transfer from PfFNR to PfFd by Alkaloids from the Stemona sp

The effect of alkaloids from the roots of* Stemona* sp. (Figure 1) on the electron transfer from* Pf*FNR to* Pf*Fd (Table 2) was investigated. In the absence of the inhibitors, the* K*_*m*_ value of* Pf*FNR for* Pf*Fd was 0.56 *μ*M, which was consistent with previous studies [28]. The inhibition assay was performed under the same conditions used in the* K*_*m*_ determination employing the following conditions: the concentration of* Pf*Fd was 1 *μ*M, the concentration of* Pf*FNR was 10 nM, and the concentration of tested compounds was 100 ppm. The % inhibition was calculated according to (1) and the results are listed in Table 2.

In this case, the same three compounds inhibited the reaction, while the two compounds exhibited a negative inhibition, but to different extents as compared to the results in Table 1. Croomine showed the largest inhibition on the reaction the FNR activity and tuberstemonine showed slight inhibition. This could be due to the inhibition of either FNR catalysis or NADPH binding to FNR. Croomine was previously shown to inhibit the activity of dihydrofolate reductase (DHFR, an enzyme for antifolate drug mechanism). The IC_50_ of croomine to the DHFR was 5.29 *μ*M, while tuberostemonine was not active to the enzyme [17]. Ramli et al. (2015) reported that isoprotostemonine and javastemonine A have low to in active against multidrug resistant K1CB1 and wild type and antifolate strains of* P. falciparum* parasites in vitro [20]. On the other hand, isoprotostemonine and javastemonine A showed negative inhibition and they accelerated FNR reaction. The javastemonine A exhibited moderate activities against* P. falciparum* TM4 and KI strains with IC_50_ of 17.7 and 16.8 ppm, respectively [20].

The diaphorase activity of* Pf*FNR in the presence of the* Stemona* alkaloids at 100 ppm is shown in Table 2. In this case, tuberostemonine alkaloids showed the highest inhibition and* epi*-croomine showed slight inhibition. Tuberostemonine could inhibit electron transfer process of* Pf*FNR to* Pf*Fd enzyme. Isoprotostemonine and javastemonine A again exhibited a negative inhibition, because both alkaloids have double bonds conjugated to carbonyl group. Isoprotostemonine and javastemonine A could be considered as trigger electron delocalization and stabilisation of resonance.

### 5.3. Docking Analysis of Alkaloids toward Protein

**Figure 3:**
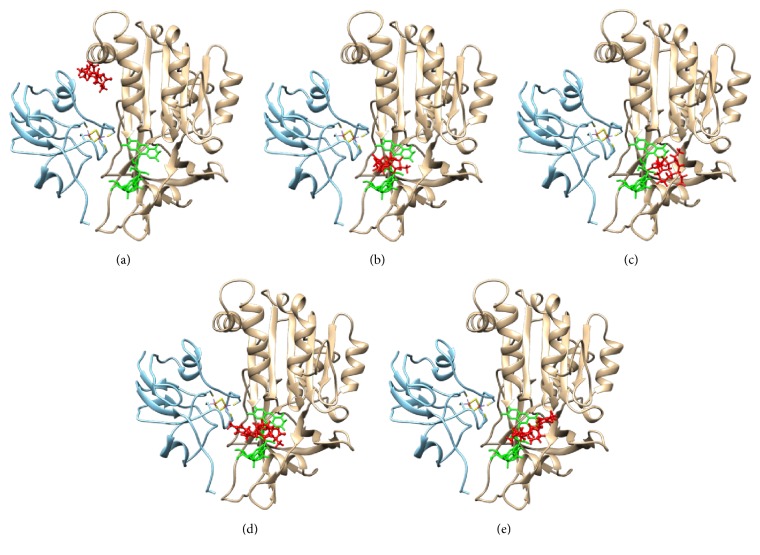
Interaction between receptors of Fd (blue), FNR (brown), and ligand (red): (a) croomine, (b)* Epi*-croomine, (c) tuberstemonine, (d) isoprotostemonine, and (e) javastemonine A.

The interaction between the alkaloids from the* Stemona *sp. and* Pf*Fd-*Pf*FNR was studied by molecular docking. The structure of* Pf*FNR was obtained from the Protein Data Bank (PDBID 1GAQ)[23]. Five alkaloids from* Stemona *sp. were expected to bind to* Pf*FNR. Figure 2 showed the location of the largest cluster of the spheres as the representation of the binding site and grid box on the receptor. This result indicated that the parameters used in the procedure could be extended to search for the binding conformation to the active site for the other ligands (alkaloids).  Figure 3 showed that interaction between receptors of* Pf*Fd (blue),* Pf*FNR (brown), and ligand (red). FAD (green) is not involved in bonding receptors-ligand.

Electron transfer is going on in* Plasmodium *from* Pf*FNR to* Pf*Fd through electrostatics interaction during respiration process [9]. Position of croomine displayed at the middle between* Pf*FNR and* Pf*Fd, while* epi*-croomine, tuberostemonine, isoprotostemonine, and javastemonine A were on the edge of the receptors (Figure 3). Croomine showed inhibition 33%, which was higher than tuberostemonine and* epi*-croomine. Croomine could obstruct electron transfer from* Pf*FNR to* Pf*Fd. This result was supported by research report foregoing that croomine inhibited competitively the human DHFR enzyme at 10 ppm [17].

Tuberostemonine is stenine alkaloid, while croomine and* epi*-croomine are spironine, and isoprotostemonine and javastemonine A are stemoamide alkaloids types [29]. The characteristic of stenine type is 4-fused rings in its structure, and spironine type has only 2-fused rings. N atom in 4-fused rings structure is rigid and stable. It is likely that the N atom in 4-fused ring is important for the diaphorase activity.

## 6. Conclusions

Croomine could inhibit the activity of FNR enzyme and electron transfer from* Pf*FNR to* Pf*Fd. Thus, croomine from* S. tuberosa* appeared to have potential as an antimalarial agent. Tuberostemonine inhibited diaphorase for redox reaction.

## Figures and Tables

**Table 1:** Inhibition (%) of *NADPH-dependent catalytic activity of PfFNR*.

Alkaloids *∗*	dA/dt*∗∗*	% Inhibition
Control	0.059	-
Croomine	0.039	33.9
Epi-croomine	0.059	0.7
Tuberstemonine	0.052	11.5
Isoprotostemonine	0.088	-48.8
Javastemonine A	0.065	-9.5

*∗*Each of alkaloids is 100 ppm; *∗∗*dA/dt is the rate of absorbance change.

**Table 2:** Inhibition of diaphorase activity of *Pf*FNR.

Alkaloids*∗*	dA/dt*∗∗*	% Inhibition
Control	0.139	-
Croomine	0.138	0.7
Epi-croomine	0.122	12.2
Tuberstemonine	0.062	55.4
Isoprotostemonine	0.17	-22.3
Javastemonine A	0.144	-3.6

*∗*Each of alkaloids is 100 ppm; *∗∗*dA/dt is the rate of absorbance change.

## Data Availability

The data used to support the findings of this study are available from the corresponding author upon request.
